# Antibacterial Prophylaxis in Emergency Surgery of Abdominal Infection

**DOI:** 10.5005/jp-journals-10018-1276

**Published:** 2019-02-01

**Authors:** Fariz H Jamalov, Rauf M Agayev, Idris T Achundov, Shahin G Huseynov, Tarana P Jamalova

**Affiliations:** Department of Surgical Disease, Azerbaijan Medical University, Baku, Azerbaijan

**Keywords:** Amoxiclav, Antibiotic prophylaxis, Emergency abdominal surgery, Purulent-septic complications.

## Abstract

The results of antibiotic prophylaxis in 148 patients with destructive acute surgical sicknesses of abdominal cavity being urgently operated in the Republican hospital of Baku city from 2011 to 2016 were analyzed. Sixty-five patients were in the basic group which had got as preoperative antibiotic prophylaxis 120-hour course of amoxiclav (amoxicillin in combination with clavulanic acid). Eighty-three patients were in the control group who have performed a surgical intervention with pre-operative 120-hour antibiotic prophylaxis by Claforan (cefotaxime) combining with Metrogel (metronidazole). it was showed that applying antibiotic prophylaxis using amoxiclav positively lowered the frequency of as postoperative purulent-septic complications as recurring operations to 8.1%.

**How to cite this article:** Jamalov FH, Agayev RM, Achundov IT, Huseynov SG, Jamalova TP. Antibacterial Prophylaxis in Emergency Surgery of Abdominal Infection. Euroasian J Hepatogastroenterol, 2018;8(2):116-120.

## INTRODUCTION

The choice of the method of antibacterial prophylaxis (ABP) for the last 50 years is an actual problem of routine and, especially emergency abdominal surgery. Moreover, in recent decades a clear trend has been observed in the growth of the number of resistant strains and the frequency of nosocomial infections, in particular, infections of the surgical intervention zone. Despite the use of new, more effective antimicrobial agents, continuous improvement, improving the quality of medical care and operational technology, the overall frequency of purulent complications in the surgical clinic does not decrease.^1-3^ According to world health organization (WHO), the lethality in the development of wound infection significantly exceeds the level (up to 10 times) with a smooth postoperative period. In surgical clinics, purulent-septic infections complicate about 30% of all surgical diseases and are the direct cause of death of every 12th patient. Lethality from infection of the surgical intervention zone in some categories of patients can reach 40%, and the cost of an antibiotics-one third of the total hospital costs for medicines. The emergence of infection in the area of surgical intervention inevitably leads to an increase of at least twice the cost of oftreating patients in surgical departments.^4-6^

The frequency of nosocomial infection varies in some limits, but ultimately it depends on the type of hospital the volume and nature of the surgical intervention, the type of basic pathology and other factors.^7,8^

The aim of our work was to develop rational schemes for perioperative antibacterial prophylaxis in emergency abdominal interventions in patients with complicated intraabdominal infection.

## MATERIALS AND METHODS OF RESEARCH

A comparative assessment of the clinical efficacy of perioperative mono-ABP by semisynthetic penicillin-amoxi-cillin 1000 mg in combination with 200 mg of clavulanic acid-amoxiclav, administered by intravenous infusion in 100 mL of normal saline for 30 minutes every 8 hours has been made.

As a comparison scheme (control), was chosen peri-operative ABP by cefotaxime at a dose of 1 g (in 20 mL saline) every 12 hours and by metronidazole at a dose of 500 mg of 100 mL of the prepared solution every 8 hours, administered by intravenous slow jet (Claforan) or a drip (Metrogel) infusion. The time of drip infusion by metronidazole was 30 to 40 minutes.

An analysis of clinical observations was carried out, including 148 patients with acute destructive surgical diseases of the abdominal cavity, who underwent emergency surgery in the Republic clinical hospital named by acad. Mir-Kasimov in the period from January 2011 to the April 2016 year. In accordance with the objectives of the study, and for the convenience of conducting a comparative evaluation, all operated patients were divided into two groups. In the first, the main group included 65 patients who underwent a 120-hour course of amoxi-clav in average therapeutic doses as perioperative ABP. Second, the control group consisted of 83 patients who underwent emergency surgery with 120-hour perioperative antibacterial prophylaxis by claforan (cefotaxime) in combination with Metrogel (metronidazole) in the average therapeutic doses indicated above.

A retrospective analysis of the study provided the opportunity to form two homogeneous groups of patients selected according to the developed inclusion criteria, relating to the age interval, gender, clinical diagnosis, the volume and type of surgery.

In the analyzed group, there were 99 (66.9%) males and 49 (33.1%) females at the age from 19 to 76 years. Compared groups of patients were homogeneous by sex composition. Both in the control group and the main proportion of women accounted for about a third of the total number of patients (p = 0.05).

The main considered acute destructive surgical disease of the abdominal organs and as a result, the patient underwent a primary emergency operation (Table 1). The perforated gastric ulcer was diagnosed in 5 (3.4%) patients, 3 of them in controls (3.6%) and 2 (3.1%) in the main group. The perforated duodenal ulcer was found in 36 (24.3%) patients, of them in 20 (24.1%) controls and 16 (24.5%) of the main group. Destructive cholecystitis was detected in 25 (17.6%) patients: in 14 (16.9) controls and the 12 (18.5%) main group.

Destructive appendicitis was diagnosed in 72 (48.6%) patients: in 41 (49.4%) controls and in 31 (47.7%) main group.

Large intestinal obstruction of tumor origin took place in 9 (6.1%) patients: in 5 (6.0%)-controls and in 4 (6.2%) main group. Statistically significant differences in the percentage of cancer of different nosologies in the compared groups were not obtained (p >0.05).

**Table 1:** **Table 1:** The character of the basic disease

		*Group of patient*											
		*Control*				*Basic*				*General*			
*Disease*		*Abs.*		*Relat.*		*Abs.*		*Relat.*		*Abs.*		*Relat.*	
Perforated ulcer stomach		3		3,6		2		3,1		5		3,4	
Perforated ulcer duodenum		20		24,1		16		24,5		36		24,3	
Destructive cholecystitis		14		16,9		12		18,5		26		17,6	
Destructive appendicitis		41		49,4		31		47,7		72		48,6	
Large intestinal obstruction		5		6,0		4		6,2		9		6,1	
Total		83		100,0		65		100,0		148		100,0	

**Table 2:** **Table 2:** Emeraencv suraerv

		*Group of patient*											
		*Control*				*Basic*				*General*			
*Operations*		*Abs.*		*Relat.*		*Abs.*		*Relat.*		*Abs.*		*Relat.*	
Distal stomach resection		3		3.6		2		3.1		5		3.4	
Suturina of perforated duodenal ulcer		20		24.1		16		24.6		36		24.3	
cholecystectomy		14		16.9		12		18.5		26		17.6	
appendectomy		33		39.8		25		38.5		58		39.2	
Drainaae of appendicular abscess		8		9.6		6		9.2		14		9.4	
Obstructive resection of the colon (operation by Hartman)		5		6.0		4		6.1		9		6.1	
Total		83		100		65		100		148		100	

## RESULTS AND DISCUSSION

Urgent interventions were performed in the study group of patients under conditions of acute purulent inflammation of the abdominal cavity, complicated by an abscess, local and widespread peritonitis. These were the so-called dirty operations, accompanied by the highest risk of purulent-septic complication reaching according to the literature, 20 to 40%. The volume of surgical intervention as a whole corresponded to the clinical diagnosis, that is, the localization and prevalence of the infectious process in the abdominal cavity. Compared groups for the nature and volume of emergency intervention were found to be homogeneous as there was no reliable difference in relative indicators for each type of operation (p = 0.05). In all observations performed: elimination of the focus of infection, sanation and drainage of the abdominal cavity, decompression of the gastrointestinal tract (Table 2).

Distal resection of the stomach was performed in 5 (3.4%) patients: in the control group-in 3 (3.6%) in the main group 2 (3.1%), the reconstruction by the method of Bilroth-1-in 3, by Bilroth- II-in 2 patients.

Suturing of perforated duodenal ulcer performed in 36 (24.3%) patients: in the control group in 20 (24.1%) in the main group in 16 (24.6%). In 20 (55.6%) patients this operation was supplemented with stem vagotomy and pyloroplasty according to the Heyneke-Mikulic.

Cholecystectomy in all 26 (17,6%) observations was performed from the medial laparotomy access: in the control group in 14 (16.9%) patients, in the main group in 12 (18.5%). At the same time, in 8 (30.8%) of them was performed external drainage of common bile duct: in 6-because of cholangitis, mechanical jaundice and choledocholithiasis confirmed during operation by cholangiography; in 2-because of bedsores of the common bile duct (vesicular-choledochal fistula). Appendectomy was performed in a typical way in 58 (39.2%) patients: in the control group in 33 (39.8%) in the main group in 25 (38.5%). In 16 (27.6%)- from the lower middle laparotomic access in connection with the clinical picture of peritonitis. In 10 out of 14 patients, appendicular infiltration with abscessing was opened and drained from extraperitoneal access by Pirogov. In 4-the oblique laparotomic access in the right iliac region by Mac-Burney. In none of the 14 observations did the vermiform appendage from infiltration not removed.

Resection of the large intestine with a tumor in conditions of obstruction was performed in 9 (6.1%) patients: in controls group-5 (6.0%) in the main-4 (6.1%). In all observations, an obstructive resection of the affected segment of the intestine was performed with the formation of the single-stemmed colostomy and suturing the distal end of the intestine.

In total, purulent-septic complications in the surgical intervention area developed in 9 ( 6.1%) patients, of them in the control group in 8 (99.6%), in the main group in 1 (1.5%). The difference between these indicators was statistically reliable (p = 0.05). Antibacterial immunopro-phylaxis with amoxiclav is accompanied by a decrease in the frequency of purulent-septic complications in the surgical intervention zone at 8.1% (Table 3).

Festering of a laparotomic wound developed in 6 (4%) patients: in 5 (6%)-in the control, in 1 (1.5%) in the main group. The intraabdominal abscess was diagnosed in 2 (1.4%) patients (both of the control group). The paraco-lostomy abscesses were formed in 1 (0.7%) patient also of the control group. It is important to note, that in the general group of patients there were no cases of continuing peritonitis.

Pulmonary infectious complications after emergency operation were recorded in 7 (4.7%) patients: of them in 5 (6.0%)-in control and 2 (3.1%)-in the main group. Statistically, the reliable difference was not observed when the frequency of pulmonary complications was compared (p = 0.05) (Table 4).

**Table 3:** **Table 3:** Structure of purulent complications of the operation zone

		*Number of complications in the group*											
		*Control (n = 83)*				*Basic (n = 65)*				*General (n = 148)*			
*Complications*		*Abs.*		*Relat.*		*Abs.*		*Relat.*		*Abs.*		*Relat.*	
Suppuration of the laparotomy wound		5		62.5		1		100		6		66.7	
Intraabdominal abscess		2		25		-		-		2		22.2	
Paracolostomy abscess		1		12.5		-		-		1		11.1	
peritonitis		-		-		-		-		-		-	
Intestinal fistula		-		-		-		-		-		-	
Total		8		100		1		100		9		100	

**Table 4:** **Table 4:** Structure of postoperative complications

		*Number of complications in the group*											
		*Control (n = 83)*				*Basic (n = 65)*				*General (n = 148)*			
*Types of complications*		*Abs.*		*Relat.*		*Abs.*		*Relat.*		*Abs.*		*Relat.*	
Thrombophlebitis		4		26.7		3		27.3		7		26.9	
Hypertensive crisis		3		20		3		27.3		6		23.1	
Cardiovascular failure		2		13.3		1		9.1		3		11.5	
Erosive gastroduodenitis		2		13.3		1		9.1		3		11.5	
Shoulder plexitis		1		6.7		1		9.1		2		7.7	
Adhesive intestinal obstruction		1		6.7		1		9.1		2		7.7	
Myocardial infarction		1		6.7		-		-		1		3.8	
Thromboembolism of pulmonary artery		1		6.7		-		-		1		3.8	
Acute psychosis		-		-		1		9.1		1		3.8	
Total		15		100		11		100		26		100	

All about the suppuration of the laparotomic wound, abscesses of the abdominal wall and abdominal cavity repeated interventions were performed in 9 (6.1%) patients, of them in 8 (9.6%) in control group (n = 83) and 1 (1.5%) in the main group (n = 65). Statistical analysis showed a reliable difference in compared relative values (p 0.05). Obviously that the use of mono ABP by amoxiclav statistically reliable reduced the frequency of postoperative purulent-septic complications and repeated operations in the main group on 8.1%. Relaparotomy was performed in 2 (2.4%) patients because of intraabdominal abscesses: in 1 patient-subhepatic abscess after cholecys-tectomy, in 1 patient-anterior (subaponeurotic) abscess in the right iliac fossa. Reliable longer (on average for 5 days), were in the hospital after emergency operations, patients of the control group, compared with patients in the main-13.2 ± 0.6 days against 8.1 ± 0.3 days (p = 0.05). The use of amoxiclav in the prevention of purulent-septic complication in emergency abdominal surgery led to a reduction in the duration of the postoperative stay in the hospital of patients with complications (n = 29) was 17.3 ± 0.8 days, and patients without complications (n = 119) - 7.2 ± 0.3 days, that is, 10 days less (p = 0.01).

Bacteriological analysis of wound discharge was performed in all 6 patients with suppuration of a lapa-rotomic wound. In all observations, the growth of pathogenic flora was obtained. It is established that mainly they were aerobes (66.7%) or their associations with anaerobes (33.7%). Among the aerobics was dominated *E. coli* (83.3%). Anaerobes were represented by bacteroids and non-differentiated spore-forming sticks. In all three inoculations of the contents of abscesses, the growth of pathogens has been obtained. In all cases, it was a mixed aerobic-anaerobic flora-an association of the *E. coli* strains with B. fragilis.

Antibacterial prophylaxis was considered effective by the clinical recovery. Antibacterial prophylaxis was considered ineffective (unsuccessful) if purulent-septic complications in the zone of surgical intervention or from the side of conjugated organs (respiratory system, etc.) were recorded during the procedure or at the end of it until the patient was discharged from the hospital.

The total frequency of postoperative pyo-septic complications in the general group was 9.5% (14 from 148 patients), in the control group-13.2% (11 from 83 patients) in the main 4.6% ( 3 from 65 patients).

Thus, the clinical efficacy of ABP in the general group (n = 148) was 90.5% at the same time it was equal to 86.8% and in the main to 95.4% (p = 0.05). It turns out that perioperative 120-hour ABP with amoxiclav had a significantly greater (by 8%) efficacy compared with 120-hour perioperative prophylaxis with cefotaxime (Claforan) in combination with metronidazole (Metrogel).

In the near postoperative period, the various options of allergic reactions and their combination were noted in 23 (15.5%) patients. A total of 33 reactions were recorded: in the main group 4 (6.1%), in control in 19 (22,9%).

Thus the frequency of allergic reactions in the control group was 16% higher (Table 5)

During ABP in 37 (25%) patients various toxic reactions to antibiotics were recorded. In the main group, they were observed only in 7 (10.8%) patients and in the control group in 30 patients (p 0.05).

Thus, it was reliably established, that a 120-hour mono ABP with amoxiclav is accompanied by a 25% lower frequency of toxic reactions compared with the traditional ABP with Claforan in combination with Metrogel (Table 6).

To a side, chemotherapeutic reactions include diarrhea, which develops due to dysbacteriosis, candidiasis (as a variant of superinfection) and pseudomembranous colitis.

**Table 5:** **Table 5:** Structure of allergic reactions

		*The number of reactions in the group*											
		*Control (n = 79)*				*Basic (n = 114)*				*General (n = 193)*			
*Reactions*		*Abs.*		*Relat.*		*Abs.*		*Relat.*		*Abs.*		*Relat.*	
Arthralgia		8				3				11			
Pruritus		6				1				7			
Convulsions		4				2				6			
Urticaria		4				1		-		5			
Exudative erythema		-		-		-		-		-		-	
Bronchospasm		-		-		-		-		-		-	
Quinckey’s edema		-		-		-		-		-		-	
Anaphylactic shock		-		-		-		-		-		-	
Total		22		100		7		100		29		100	

**Table 6:** **Table 6.** Structure of toxic reactions

		*The number of reactions in the group*											
		*Control (n = 79)*				*Basic (n = 114)*				*General (n = 193)*			
*Reactions*		*Abs.*		*Relat.*		*Abs.*		*Relat.*		*Abs.*		*Relat.*	
Neutropenia (<70%)		11		25.6		3		20		14		24.1	
Headache		5		11.6		5		33.3		10		17.2	
Transaminase elevation		8		18.6		2		13.3		10		17.2	
Nausea		7		16.3		2		13.3		9		15.5	
Albuminuria		7		16.3		2		13.3		9		15.5	
Dizziness		3		7.0		1		6.8		4		6.9	
Eosinophilia (>4%)		2		4.6		-		-		2		3.4	
Vomiting		-		-		-		-		-		-	
Visual impairment		-		-		-		-		-		-	
Leukopenia (< 4 × 103)		-		-		-		-		-		-	
Thrombocytopenia (<80×103)		-		-		-		-		-		-	
Increased alkaline phosphatase		-		-		-		-		-		-	
Bilirubinemia (general >17 mmol/L)		-		-		-		-		-		-	
Increased creatinine		-		-		-		-		-		-	
Hematuria		-		-		-		-		-		-	
Total		43		100		15		100		58		100	

A total of these adverse chemotherapeutic reactions were noted in 23 (15.5%) patients, of them in 5 (7.7%) patients of the main group, and in 18 (21.7%) in the control group (p 0.05) (Table 7).

Thus, it was reliably established, that a 120-hour ABP with amoxiclav is accompanied lower (by a 14%) frequency aside chemotherapeutic reactions compared with 120-hour prophylaxis with claforan in combination with Metrogyl.

A total of 23 patients registered 31 chemotherapeutic reactions to antibiotics: 2 reactions-in 8, 1 reaction in 15 patients.

## CONCLUSION

The 120-hour monoantibiotic prophylaxis with amoxiclav in comparison with the same duration of prophylaxis with Claforan in combination with Metrogyl allows to reduce the duration of stay of patients in the hospital, on average, by 5 days the frequency of allergic reactions by 16%, toxic reactions by 25% and undesirable chemothera-peutic reactions by 14% with a consistently high level of clinical efficacy to prevent purulent-septic complications, which is 95,4%.

**Table 7:** **Table 7:** Structure of adverse chemotherapeutic reactions

		*The number of reactions in the group*											
		*Control (n = 79)*				*Basic (n = 114)*				*General (n = 193)*			
*Reaction*		*Abs.*		*Relat.*		*Abs.*		*Relat.*		*Abs.*		*Relat.*	
Diarrhea		16		57.1		7		70.0		23		60.5	
Candidiasis		12		42.9		3		30.0		15		39.5	
Pseudomembranous colitis		-		-		-		-		-		-	
Total		28		100		10		100		38		100	

## References

[1] Akhundov IT, IbishovSh F, Alekperov DA (2000). The endolymphatic immunomodulating therapy in correction of immune status disturbances in patients with peritonitis. // 5-th World Congress on Trauma, Shock, Inflammation and Sepsis:.

[2] Bratzler DW, Houck PM (2004). Antimicrobial prophylaxis for surgery: Anadvisory statement from the national surgical infection preventionproject. //Clinical Infectious Diseases.

[3] Mui LM, Ng CSH, Wong SKH, Lam YH, Fung TMK, Fok KL (2005). Optimum duration of prophylactic antibiotics in acute non-perforated appendicitis. //ANZ Journal of Surgery.

[4] Esposito S, Noviello S, Vanasia A, Venturino P (2004). Ceftriaxone versusOther Antibiotics for Surgical Prophylaxis: A Meta-Analysis. //Clinical Drug Investigation.

[5] Pratt RJ, Pellowe CM, Wilson JA, Loveday HP, Harper PJ, Jones SR (2007). epic 2: National Evidence-Based Guidelines for PreventingHealthcare-Associated Infections in NHS Hospitals in England. //JHosp Infect.

[6] Weber WP, Marti WR, Zwahlen M, Misteli H, Rosenthal R, Reck S (2008). The timing of surgical antimicrobial prophylaxis. //Ann Surg.

[7] Bratzler DW, Dellinger EP, Olsen KM, Perl TM, Auwaerter PG, Bolon MK (2013). Clinical practice guidelines for antimicrobial prophylaxis in surgery. //Am J Health Syst Pharm.

[8] David N, Gilbert HF, Chambers GM, Eliopoulos MS (2015). The Sanford Guide to Antimicrobial Therapy // Antimicrobial Therapy.

